# 

**Published:** 1873-05-15

**Authors:** 


### The Medical Examiner.

A Semi-Monthly Journal of Medical Sciences.

No. 10. CHICAGO, MAY 15, 1873. Vol. XIV.

TERMS.—Yearly Subscriptions, Three Dollars, in advance. Single Numbers Fifteen Cents.
All Communications should be addressed to Publishers Medical Examiner, 792 Wabash Av., Chicago.

